# Effect of mass dihydroartemisinin–piperaquine administration in southern Mozambique on the carriage of molecular markers of antimalarial resistance

**DOI:** 10.1371/journal.pone.0240174

**Published:** 2020-10-19

**Authors:** Himanshu Gupta, Beatriz Galatas, Arlindo Chidimatembue, Silvie Huijben, Pau Cisteró, Gloria Matambisso, Lidia Nhamussua, Wilson Simone, Quique Bassat, Didier Ménard, Pascal Ringwald, N. Regina Rabinovich, Pedro L. Alonso, Francisco Saúte, Pedro Aide, Alfredo Mayor

**Affiliations:** 1 ISGlobal, Barcelona Institute for Global Health, Hospital Clínic—Universitat de Barcelona, Barcelona, Spain; 2 Centro de Investigação em Saúde de Manhiça, Manhica, Mozambique; 3 ICREA, Pg. Lluís Companys, Barcelona, Spain; 4 Spanish Consortium for Research in Epidemiology and Public Health (CIBERESP), Madrid, Spain; 5 Pediatric Infectious Diseases Unit, Pediatrics Department, Hospital Sant Joan de Déu (University of Barcelona), Barcelona, Spain; 6 Institut Pasteur, Paris, France; 7 INSERM U1201, Paris, France; 8 CNRS ERL9195, Paris, France; 9 World Health Organization (WHO), Global Malaria Programme, Geneva, Switzerland; 10 Harvard T.H. Chan School of Public Health, Boston, Massachusetts, United States of America; 11 National Institute of Health, Ministry of Health, Manhica, Mozambique; Instituto Rene Rachou, BRAZIL

## Abstract

**Background:**

Mass drug administration (MDA) can rapidly reduce the burden of *Plasmodium falciparum* (*Pf*). However, concerns remain about its contribution to select for antimalarial drug resistance.

**Methods:**

We used Sanger sequencing and real-time PCR to determine the proportion of molecular markers associated with antimalarial resistance (*k13*, *pfpm2*, *pfmdr1 and pfcrt*) in *Pf* isolates collected before (n = 99) and after (n = 112) the implementation of two monthly MDA rounds with dihydroartemisinin–piperaquine (DHAp) for two consecutive years in Magude district of Southern Mozambique.

**Results:**

None of the *k13* polymorphisms associated with artemisinin resistance were observed in the *Pf* isolates analyzed. The proportion of *Pf* isolates with multiple copies of *pfpm2*, an amplification associated with piperaquine resistance, was similar in pre- (4.9%) and post-MDA groups (3.4%; p = 1.000). No statistically significant differences were observed between pre- and post-MDA groups in the proportion of *Pf* isolates neither with mutations in *pfcrt* and *pfmdr1* genes, nor with the carriage of *pfmdr1* multiple copies (p>0.05).

**Conclusions:**

This study does not show any evidence of increased frequency of molecular makers of antimalarial resistance after MDA with DHAp in southern Mozambique where markers of antimalarial resistance were absent or low at the beginning of the intervention.

## Introduction

The administration of drugs to whole populations irrespective of disease status aims to prevent and reduce morbidity on the one hand and reduce transmission on the other, altogether improving global health [1]. This strategy, known as mass drug administration (MDA), is recommended by the World Health Organization (WHO) to control or eliminate several neglected tropical pathogens, including bacteria and helminths. Recent studies suggest that MDA, when used as part of a comprehensive and well-organized elimination programme, can be a useful additional tool to accelerate the path towards malaria elimination [2–6].

The selection and subsequent spread of drug resistance is a major concern when administering any antimicrobial agent on a mass scale, especially if the pathogen is being targeted with only a single drug [1]. However, evidence for the establishment of drug resistance at large scale driven by MDA is limited, in part due to the scarcity of programmes which have monitored changes in drug efficacy or potential drug resistance [1]. Few reports have shown emergence of azithromycin-resistant *Treponema pallidum* [7] and temporary increases in carriage of macrolide-resistance following azithromycin MDAs [8,9], although no evidence of drug resistance has been documented after long-term MDA in other situations [1,10]. With regards to anti-malarial MDAs, circumstantial evidence has linked indirect MDA using medicated salts to the emergence of chloroquine (CQ) resistance in the 1980s [5].

Concerns remain as to whether current MDA strategies based on the use of artemisinin-based combination therapies (ACT) might contribute to the emergence and spread of antimalarial drug resistance [4,5]. MDA leads to opposing forces on the selection for resistance and it is not at all that obvious that MDA would always lead to resistance evolution. On the one hand, MDA drastically decreases total number of malaria parasites, which reduces to probability of *de novo* resistance mutations. However, it would also maximize selective pressure for any resistant mutant that exists or does arise [11]. How both these forces play out in different epidemiological contexts is not yet known. Mathematical models have estimated that the implementation of MDA with atovaquone–proguanil would lead to rapid selection for high-level resistance, even after a single round of MDA [12]. Modeling predictions suggested reduction in the effectiveness of subsequent rounds of treatment, with total loss of efficacy within 4–5 years, although mutations in *cytochrome b* gene were later found to be lethal in the mosquito host [13] and thus to impede transmission of resistant parasites. The same models suggested a lower risk in the selection of drug resistance by ACTs such as dihydroartemisinin-piperaquine (DHAp), due to the weaker resistance phenotype resulting from artemisinin resistant mutations known to date [14–16].

Several polymorphisms in the *kelch13* (*k13*) propeller gene of *P*. *falciparum* have been associated with artemisinin resistance [14–17]. The reduced copy number of *P*. *falciparum* multidrug resistance 1 (*pfmdr1)* gene has also been linked to increased sensitivity to artemisinin of trophozoite stages [18], on the contrary, multiple *pfmdr1* copies associated with declining efficacy of mefloquine–artesunate in the Thai–Myanmar border [19]. Recently, *plasmepsin*-*2* (*pfpm2*) copy number and polymorphisms in *P*. *falciparum* chloroquine resistance transporter (*pfcrt)* gene (H97Y, C101F, F145I, M343L or G353V) have been associated with decreased piperaquine sensitivity and high DHAp treatment failure rates in settings where artemisinin resistance is common [20–25]. In addition, evidence suggests that the presence of 86N and 184F alleles in *pfmdr1* gene may reduce the susceptibility to piperaquine in parasites expressing the CVIET haplotype in the *pfcrt* gene [26].

A before-after study was conducted in southern Mozambique to evaluate the impact of a package of interventions with the aim to interrupt *Plasmodium falciparum* (*Pf*) malaria transmission [27]. Two rounds of MDA with DHAp per year over 2015 and 2017, together with annual indoor residual spraying (IRS), programmatically distributed long-lasting insecticide treated nets (LLINs) and standard case management, lead to a 71.3% reduction of all-age parasite prevalence by rapid diagnostic test (from 9.1% to 2.6%), and a 61.5% reduction in case incidence at health facility level (from 195 to 75 cases per 1000). Here we aimed to determine the effect of MDA with DHAp on the selection of dihydroartemisinin and piperaquine resistance at the population level. To achieve this, we assessed molecular markers of resistance (*k13*, *pfmdr1*, *pfcrt* and *pfpm2*) among the circulating parasite population before and after the implementation of MDA with DHAp in Magude district in southern Mozambique.

## Material and methods

### Study site

Two MDA rounds separated by a period of 4–6 weeks were deployed at the beginning of the rainy seasons of 2015–16 (November and January-February) and 2016–17 (December and January-February) in Magude District (Southern Mozambique). The MDAs were targeted to the entire population of Magude (48,488 residents, 10965 households in 2015) and reached 72.3%, 58%, 66.6% and 64.8% of the population in each of the four rounds, respectively [27].

### Sample collection and study design

Blood samples were collected on Whatman 3mm filter papers from a random subsample of individuals during the first MDA round in November 2015 prior to drug administration (n = 6858) as well as from 3752 of the 3865 individuals participating in an age-stratified cross-sectional survey conducted during May 2017 (after the four rounds of MDA) among all-age individuals randomly selected from the Magude population census. qPCR was conducted on a random selection of 1271 samples collected in November 2015 and in all samples collected during May 2017.

### DNA extraction and *P*. *falciparum* detection by real time quantitative PCR (qPCR)

DNA was extracted from a half cut of the dried blood spot (corresponding to approximately 25μL blood) by using a QIAamp DNA Mini kit (Qiagen), as per the manufacturer's instructions, with a final elution in 100μL of elution buffer. The ABI PRISM 7500 HT Real-Time System (Applied Biosystems) was used to amplify purified parasite DNA templates, using a previously described method that targets the 18S rRNA gene [28,29]. A standard curve was prepared from a synchronized *in vitro* culture of 3D7 strain containing known numbers of ring-infected erythrocytes and performed in triplicate for each test with five serially diluted points. *P*. *falciparum* parasitemia was quantified in the samples by interpolating the Ct values against the standard curve. DNAs extracted from post-MDA samples with low parasitemia (less than 5 parasites/μl) were pre-amplified using PicoPLEX™ WGA Kit as per the manufacturer's instructions (Rubicon Genomics). Amplified samples were not used for copy number estimations using qPCR. PCR amplification and Sanger sequencing were performed on 21 randomly selected samples before and after pre-amplification, to confirm that the pre-amplification step was not affecting allele frequencies obtained by Sanger sequencing.

### Analysis of molecular markers of antimalarial resistance

To assess polymorphisms in *k13*, *pfcrt* and *pfmdr1* genes, purified DNA templates were amplified using 2720 Thermal Cycler (Applied Biosystems) following protocols described elsewhere for *k13* (aminoacids 427–709 of 3D7 [30,31]), *pfcrt* (aminoacids 35–120 [30]), and *pfmdr1* (two separate fragments covering aminoacids 45–209 and 984–1277 [30]) and sent to Genewiz for bi-directional sequencing. Six positive controls with known *k13* alleles and four parasite lines (3D7, 7G8, Dd2 and V1/S) with known *pfcrt* and *pfmdr1* alleles [30], as well as a negative control (water instead of template DNA), were also included. The variations in the test sequences of *k13*, *pfcrt* and *pfmdr1* were identified by sequence alignment against PF3D7_1343700, PF3D7_0709000 and PF3D7_0523000 reference sequence of 3D7 respectively, retrieved from PlasmoDB. Polymorphisms were confirmed in both sequences obtained using forward and reverse primers. Isolates with mixed alleles were considered as mutant-type for the purposes of polymorphism frequency estimation.

### *pfpm2* and *pfmdr1* copy number

A qPCR was used to assess variations in the copy number of *pfpm2* and *pfmdr1* genes as described elsewhere [30]. For each run, the *pfpm2* and *pfmdr1* copy numbers of each sample were measured in triplicate and the *pfβ-tubulin* gene was used as an endogenous control. The PCR efficiencies of the *pfpm2*, *pfmdr1* and *pfβ-tubulin* genes were measured using ten-fold dilutions of 3D7 DNA. The specificity of three primer pairs against human gDNA was also determined. Along with no template control, positive controls with the known copies (3–4) of *pfpm2* [30] and *pfmdr1* (Dd2 parasite line) were also included. All samples with C_t_ >35 for *pfpm2*, *pfmdr1* and *pfβ-tubulin* were not considered for the copy number analysis. The copy number of *pfmdr1* and *pfpm2* genes was estimated as described previously [23]. Samples with estimated copy number values above 1.5 were defined as having multiple copies and confirmed in independent qPCRs [30].

### Data analysis

Sex and residence area of pre- and post-MDA study participants were compared by Fisher’s exact test, while age and log-transformed qPCR parasite densities were compared using Student t test. The proportion of mutant alleles for each specific gene was calculated based on the frequencies of samples with wild-type and mutant alleles. The percentage of isolates with multiple copy numbers was also determined. Fisher’s exact test was used to compare the proportion of *Pf* isolates with resistant genotypes, as well as with multiple gene copy number, before and after MDA. The statistical significance was defined as a p-value<0.05.

### Ethics

Study ethical approval was obtained from the National Mozambican Ethical Review Committee (Mozambique), pharmaceutical department of the MoH (IRB), and Hospital Clínic (Barcelona, Spain) ethics review committees, and signed written informed consent was obtained from all participants or from guardians/parents in the case of minors.

## Results

### Study participants and samples analyzed

Dried blood spots (DBS) were collected from a random selection of consenting MDA1 participants and from individuals included in an age-stratified community-based cross-sectional survey, conducted in May 2017 (three months after MDA4 round). A further random selection of 1271 (November 2015) and 3752 (May 2017) DBS analyzed by qPCR targeting *Pf* 18S rRNA identified 168 and 139 *Pf* infections, respectively. Among these, samples which successfully amplified *k13* gene (99 and 112 pre- and post-MDA, respectively) were further selected for the analysis of molecular markers of resistance.

DNAs extracted from samples with low parasitemia (less than 5 parasites/μl, n = 29) in post-MDA isolates, were pre-amplified using PicoPLEX™ WGA Kit before PCR amplification of targeted genes. Allele frequencies were similar in the 21 *Pf* isolates that were tested with and without pre-amplification, meaning that pre-amplification was not affecting allele frequencies (S1 Fig). Study participants pre- and post-MDA were similar in area of residence, age, sex and qPCR-determined *Pf* densities (S1 Table).

### *k13* polymorphisms

**Fig 1:**
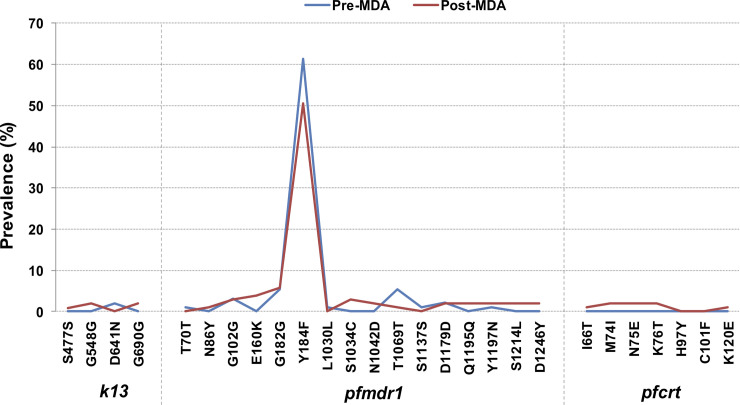
Distribution of *Plasmodium falciparum k13*, *pfmdr1* and *pfcrt* polymorphism frequencies among pre- and post-MDA isolates.

The sequences of *k13* were successfully determined in 99 (100%) and 107 (96%) pre- and post-MDA isolates, respectively. As expected, all positive controls sequencing analysis revealed the existence of wild-type and mutant alleles of *k13* polymorphisms. *k13* polymorphisms reported in Cambodian isolates [15] were absent in the *Pf* isolates analyzed in this study. However, 2 novel synonymous polymorphisms were observed at amino acid positions 477 (0.9% [1/107]) and 548 (1.9% [2/107]), as well as a non-synonymous mutation from “aspartic acid” to “asparagine” at codon 641 (2% [2/99]). The polymorphism G690G previously described in *Pf* field isolates collected in 2015 from Mozambique [30] was also observed in the studied isolates (1.9% [2/107]). No statistically significant difference was noticed when the frequency of polymorphisms was compared between pre- and post-MDA groups (p>0.05; Table 1; Fig 1).

**Table 1:** Proportion of *P*. *falciparum* isolates with *k13* gene polymorphisms in pre- and post-MDA groups.

	Pre-MDA (n = 99)	Post-MDA (n = 107)	
	n(%)	n(%)	p*
**S477S**	0 (0)	1 (0.9)	1.000
**G548G**	0 (0)	2 (1.9)	0.498
**D641N**	2 (2)	0 (0)	0.230
**G690G**	0 (0)	2 (1.9)	0.498

* Fisher’s exact test.

### *pfmdr1* polymorphisms

The sequences of *pfmdr1* were successfully determined in 93 (94%) and 105 (94%) pre- and post-MDA isolates, respectively. All positive controls sequencing analysis revealed the existence of *pfmdr1* wild- and mutant-type alleles. Nine polymorphisms (2 [22%] non-synonymous) in pre-MDA and thirteen polymorphisms (8 [61.5%] non-synonymous) in post-MDA groups were identified in the *pfmdr1* gene (Table 2). Y184F was the most common polymorphism, observed at a frequency of 61.3% (57/93) and 50.5% (53/105) in pre- and post-MDA samples, respectively (p = 0.152). The rest of non-synonymous mutations were at frequencies less than 5%, with no evidence of statistically significant differences between pre- and post-MDA groups (Table 2; Fig 1).

**Table 2:** Proportion of *P*. *falciparum* isolates with *pfmdr1* gene polymorphisms in pre- and post-MDA groups.

	Pre-MDA (n = 93)	Post-MDA (n = 105)	
	n(%)	n(%)	p*
**T70T**	1 (1.1)	0 (0)	0.470
**N86Y**	0 (0)	1 (1.0)	1.000
**G102G**	3 (3.2)	3 (2.9)	1.000
**E160K**	0 (0)	4 (3.8)	1.000
**G182G**	5 (5.4)	6 (5.7)	1.000
**Y184F**	57 (61.3)	53 (50.5)	0.152
**L1030L**	1 (1.1)	0 (0)	0.470
**S1034C**	0 (0)	3 (2.9)	0.249
**N1042D**	0 (0)	2 (1.9)	0.499
**T1069T**	5 (5.4)	1 (1.0)	0.101
**S1137S**	1 (1.1)	0 (0)	0.470
**D1179D**	2 (2.2)	2 (1.9)	1.000
**Q1195Q**	0 (0)	2 (1.9)	0.499
**Y1197N**	1 (1.1)	2 (1.9)	1.000
**S1214L**	0 (0)	2 (1.9)	0.499
**D1246Y**	0 (0)	2 (1.9)	0.499

* Fisher’s exact test.

### *pfcrt* polymorphisms

The sequences of *pfcrt* were successfully determined in 88 (89%) and 104 (93%) pre- and post-MDA isolates, respectively. Wild-type and mutant *pfcrt* polymorphisms present in positive controls were successfully detected. All *Pf* isolates carried wild-type allele at codon 72 (C), 74 (M), 75 (N), 76 (K), 97 (H) and 101 (C) in pre-MDA isolates. *P*. *falciparum* isolates collected post-MDA presented M74I, N75E and K76T polymorphisms along with newly identified I66T and K120E polymorphisms with allele frequencies <2%. No statistically significant differences were observed in polymorphism frequencies between pre- and post-MDA groups (Table 3; Fig 1).

**Table 3:** Proportion of *P*. *falciparum* isolates with *pfcrt* gene polymorphisms in pre- and post-MDA groups.

	Pre-MDA (n = 88)	Post-MDA (n = 104)	
	n(%)	n(%)	p*
**I66T**	0 (0)	1 (1.0)	1.000
**M74I**	0 (0)	2 (1.9)	0.501
**N75E**	0 (0)	2 (1.9)	0.501
**K76T**	0 (0)	2 (1.9)	0.501
**H97Y**	0 (0)	0 (0)	NA
**C101F**	0 (0)	0 (0)	NA
**K120E**	0 (0)	1 (1.0)	1.000

* Fisher’s exact test; NA–not applicable.

### *P*. *falciparum* gene copy number

**Fig 2:**
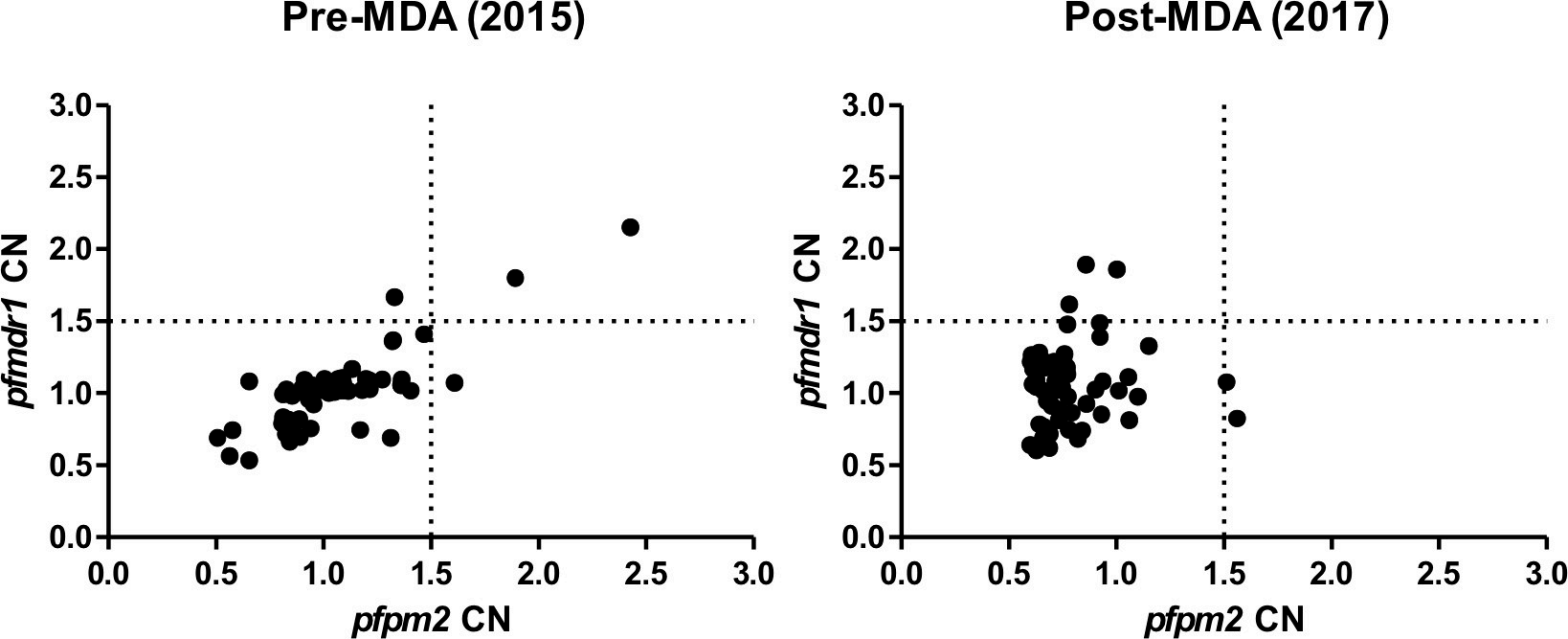
Copy number of *pfpm2* and *pfmdr1* genes in *P*. *falciparum* isolates from pre- and post-MDA groups.

Sixty-one (62%) pre-MDA and 59 (53%) post-MDA isolates were successfully analyzed for copy number variation of *pfpm2* and *pfmdr1* genes. All positive control copy numbers for *pfpm2* and *pfmdr1* genes were estimated between 3–4 copies. The lowest to highest range of estimated copy numbers were 0.61 to 2.4 (*pfpm2*) and 0.63 to 2.1 (*pfmdr1*) for pre-MDA isolates, and 0.59 to 1.56 (*pfpm2*) and 0.60 to 1.89 (*pfmdr1*) for post-MDA isolates. Using a copy number threshold of 1.5 to define multiple gene copies isolates [30], 3/61 (4.9%) and 2/59 (3.4%) of *Pf* isolates had multiple copies of *pfpm2* in pre- and post-MDA isolates, respectively (Fig 2). Similarly, 3/61 (4.9%) and 4/59 (5.1%) of *Pf* isolates had multiple copies of *pfmdr1* in pre- and post-MDA isolates, respectively (S2 Table). No statistically significant differences were noticed in the proportion of *Pf* isolates with multiple copies of *pfpm2* (p = 1.000) and *pfmdr1* (p = 1.000) between pre- and post-MDA groups.

## Discussion

Although the use of MDA as a tool to rapidly reduce malaria transmission has become increasingly more popular [4,32–36], the question remains as to whether the distribution of antimalarial drugs at a population level could lead to an increase in drug resistance [37]. This study revealed that there was no emergence of drug resistance after the implementation of four MDA rounds with moderate coverage levels (58–72%). Therefore, there is no evidence that effectively implemented MDA with an ACT in low- to moderate-endemic settings will lead to drug resistance, and therefore supports the use of DHAp as a drug for future MDAs in areas with no detectable markers of resistance at the start of the intervention.

In line with previous studies conducted in Mozambique [30], polymorphisms in *k13* gene associated with artemisinin resistance were not found in the *Pf* isolates collected in Magude district, while the proportion of multiple *pfpm2* copies was below 5% (4.9% before MDA and 4.3% post-MDA). Other markers of piperaquine resistance in *pfcrt* gene (present in the 35–120 aminoacid region of the protein) were completely absent in these isolates. The absence or low levels of molecular markers of parasite resistance against artemisinin and piperaquine suggests good efficacy of these drugs against *Pf* isolates circulating in the study area. This is aligned with the substantial reduction in malaria incidence achieved with the MDA rounds conducted in Magude, together with annual indoor residual spraying, programmatically distributed long-lasting insecticide treated nets and standard case management [27]. Although comforting at this stage, piperaquine resistance may spread in Mozambique if piperaquine drug pressure increases and artemisinin resistance emerges, subsequently facilitating selection of resistance to ACT partner drugs, as observed in Southeast Asia [23,38,39]. However, no statistically significant differences in the proportion of *k13*, *pfcrt* and *pfmdr1* polymorphisms and multiple copies of *pfpm2* and *pfmdr1* genes were observed between pre- and post-MDA *Pf* isolates.

These results are in line with reports from other settings where artemisinin and piperaquine resistance is higher compared to Mozambique. Data from Myanmar show a stable proportion of *k13* wild-type parasites and no piperaquine resistance during a three-year period in which intense DHAp deployment for targeted MDA substantially decreased malaria incidence [40]. Similarly, no statistical differences were observed in the proportion of *k13* polymorphisms in *Pf* isolates collected before and after 3 monthly MDA rounds in Comoros [32]. In Cambodia, transmission of multidrug-resistant *Pf* parasites was interrupted using MDA with high coverage [36], and no reports of clinical cases were reported for at least 1 year. Similarly, other community-based trials of MDA with DHAp have demonstrated this drug to be efficacious, effective and safe in killing malaria parasites in pre-elimination settings [2,33,35,41,42], without any suggestion of decreased effectiveness due to expansion of antimalarial resistance. A recent study in South-East Asia has also shown that DHAp mass treatments have not selected resistance further in areas with high frequencies of parasites carrying mutations associated with DHAp resistance [43], thus supporting the use of targeted mass treatment. To our knowledge, recent studies describing MDA with DHAp implemented in Africa have not assessed the molecular markers of DHAp resistance [44]. In contrast to the lack of studies reporting the molecular markers of DHAp resistance in Africa, seasonal malaria chemoprevention trial has reported the frequency of molecular markers of sulfadoxine-pyrimethamine (SP) plus amodiaquine (AQ) resistance, which did not increase significantly over the study period [45].

The absence of a signature of resistance evolution following MDA with DHAp in southern Mozambique and in other endemic settings could be explained by several factors. First, initial signals of artemisinin resistance characterized by slow parasite clearance [46] before the appearance of *in vivo* resistance [47] may lower the probability of successful transmission of a resistant parasite strains present in a population receiving MDA. This transmission may be further reduced by strong vector control approaches. Second, the administration of drugs with a fairly short half-life, such as derivatives of artemisinin, have lower risk compared to longer acting drugs of selecting resistant parasites, as subtherapeutic concentrations are expected to be available for the parasites for only a limited period of time. Additionally, although MDA will expose many asymptomatic infections to the antimalarial used, the low parasite burdens and the effective host defense mechanisms in these subclinical carriers will reduce the risk of emergence of resistance and the transmission potential of any recurrent infection [11]. Fourth, the homogeneity in the population’s drug concentration profiles after MDA, which is given to everyone at the same time, reduces the opportunity for selection of a higher level of resistance as parasites are less likely to encounter high drug concentrations in the next host. Lastly, MDA decreases the incidence of symptomatic malaria in the overall population, which results in less use of the antimalarial, lower parasite biomass exposed to the antimalarials and thus fewer opportunities for the selection of drug resistance.

Mathematical modeling suggests that the spread of drug resistance is strongly dependent on treatment coverage, whereby initial allele frequencies and frequency of treatment play key roles as well [48]. Poor coverage, poor adherence or substantial migration, and therefore reduced effectiveness of MDA, will probably increase the probability of selecting resistance. The risk of selecting resistance may be likely reduced if MDAs are deployed when the parasite biomass is at the lowest levels (i.e., during the dry season in areas of low seasonal transmission) and when there is good adherence to the treatment provided. Therefore, areas with malaria elimination strategies in place that reduces malaria transmission, and hence immunity [49], may be are more susceptible to a rapid emergence and spread of resistant parasites [50]. In this situation, MDA could select for resistant mutants that are introduced in the population (such as migration). Further studies would be needed to understand if parasite adaptations and compensatory responses to stress situations driven by steep reductions in malaria transmission, such as increased parasite investment on gametocyte production to maximize transmission [51,52], or reductions in the intensity of between-genotype competition within hosts [53], may have a long-term impact on the emergence and spread of antimalarial resistance.

The results of this study are subject to several limitations. First, there is a possibility the intervention did lead to some evolution of resistance, but this was too rare to be detected with the relatively low number of samples analyzed in this study. Furthermore, the presence of multiple clones in each infection may limit the chances to detect mutant alleles is these constitute a minor fraction in the infection. However, the description of DHAp molecular markers will provide the baseline information to identify the potential expansion of resistant parasites if malaria resurges in the area. Second, this study may have missed the emergence of mutations at densities below the sensitivity limit of the PCRs and Sanger sequencing assays. Third, DNA degradation during sample storage, processing, freezing and thawing could potentially have biased the assays towards the detection of wild type polymorphisms if parasites carrying mutations associated with resistance are more frequently being degraded due to their lower densities. Fourth, our sequencing approach [30] did not include polymorphisms in codons 145, 343 or 353 of *pfcrt*, which have been associated with decreased piperaquine sensitivity and high DHAp treatment failure rates in settings where artemisinin resistance is common [20–25]. Lastly, we cannot discard the possibility of emerging resistance following MDA if conducted on a much larger scale, although this study demonstrates the likelihood of such an event happening is relatively low.

Overall, this study shows that in sub-Saharan setting with absent or low background levels of artemisinin and piperaquine resistance, two monthly MDA rounds with DHAp moderate coverage levels for two consecutive years would not increase the frequency of molecular makers of antimalarial resistance in the general *Pf* parasite population. Based on the findings of the present study and other previous studies, there is no suggestion that a well-implemented MDA with an ACT has led to drug resistance [3,5]. Therefore, DHAp proves to be safe and effective tool in reducing the malaria burden [2,35,36,40,54] and could be useful for future MDAs following WHO recommendations for MDA implementation. Enabling good molecular surveillance systems should be a prerequisite for the use of community-wide distribution of antimalarials aiming the interruption of malaria transmission.

## Supporting information

S1 Fig Sequencing results of *P*. *falciparum* samples before (A) and after (B) pre-amplification.
(PDF)

S1 Table Main characteristics of study participants. (PDF)

S2 Table Copy number of *pfpm2* and *pfmdr1* in *P*. *falciparum* isolates from pre- and post-MDA groups. (PDF)
