# Supplementary material for: Effect of mass dihydroartemisinin–piperaquine administration in southern Mozambique on the carriage of molecular markers of antimalarial resistance
Source: PLoS One. 2020 Oct 19;15(10):e0240174. doi: 10.1371/journal.pone.0240174 (PMC7571678; doi:10.1371/journal.pone.0240174)
Supplement: S1 Fig — Sequencing results of P. falciparum samples before (A) and after (B) pre-amplification. (PDF) [file pone.0240174.s001.pdf]

**S1 Fig.** Sequencing results of *P. falciparum* samples before (A) and after (B) pre-amplification.

**A**

| Sample ID | *pfcrt* I66T | *pfcrt* M74I | *pfcrt* N75E | *pfcrt* K76T | *pfcrt* K120E | *K13* S477S | *pfmdr1* T1069T | *pfmdr1* Q1195Q | *pfmdr1* S1214L | *pfmdr1* D1246Y |
|---|---|---|---|---|---|---|---|---|---|---|
| 17PM27 | M | W | W | W | M | W | W | W | W | W |
| 17PM68 | W | W | W | W | W | W | W | M | M | W |
| 17PM72 | W | W | W | W | W | W | W | W | W | W |
| 17PM73 | W | W | W | W | W | W | W | W | W | W |
| 17PM74 | W | W | W | W | W | W | W | W | W | W |
| 17PM76 | W | W | W | W | W | W | M | W | W | W |
| 17PM77 | W | W | W | W | W | W | W | W | W | W |
| 17PM78 | W | W | W | W | W | W | W | W | W | W |
| 17PM94 | W | W | W | W | W | W | W | W | W | W |
| 17PM96 | W | W | W | W | W | W | W | W | W | W |
| 17PM103 | W | W | W | W | W | W | W | W | W | W |
| 17PM105 | W | W | W | W | W | W | W | W | W | W |
| 17PM111 | W | W | W | W | W | W | W | W | W | W |
| 17PM117 | W | W | W | W | W | M | W | W | W | W |
| L058 | W | M | M | M | W | W | W | W | W | W |
| L060 | W | M | M | M | W | W | W | W | W | W |
| L111 | W | M | M | M | W | W | W | W | W | W |
| L198 | W | M | M | M | W | W | W | W | W | W |
| L214 | W | M | M | M | W | W | W | W | W | W |
| L225 | W | M | M | M | W | W | W | W | W | M |
| L231 | W | M | M | M | W | W | W | W | W | W |

M = Mutant allele W = Wild-type allele MD = Missing Data

**B**

| Sample ID | *pfcrt* I66T | *pfcrt* M74I | *pfcrt* N75E | *pfcrt* K76T | *pfcrt* K120E | *K13* S477S | *pfmdr1* T1069T | *pfmdr1* Q1195Q | *pfmdr1* S1214L | *pfmdr1* D1246Y |
|---|---|---|---|---|---|---|---|---|---|---|
| 17PM27 | M | W | W | W | M | W | W | W | W | W |
| 17PM68 | W | W | W | W | W | W | W | M | M | W |
| 17PM72 | W | W | W | W | W | W | W | W | W | W |
| 17PM73 | MD | MD | MD | MD | MD | W | MD | MD | MD | MD |
| 17PM74 | W | W | W | W | W | W | W | W | W | W |
| 17PM76 | W | W | W | W | W | W | M | W | W | W |
| 17PM77 | W | W | W | W | W | W | W | W | W | W |
| 17PM78 | W | W | W | W | W | W | W | W | W | W |
| 17PM94 | W | W | W | W | W | W | W | W | W | W |
| 17PM96 | W | W | W | W | W | W | W | W | W | W |
| 17PM103 | W | W | W | W | W | W | W | W | W | W |
| 17PM105 | W | W | W | W | W | W | W | W | W | W |
| 17PM111 | W | W | W | W | W | W | W | W | W | W |
| 17PM117 | W | W | W | W | W | M | W | W | W | W |
| L058 | W | M | M | M | W | W | W | W | W | W |
| L060 | W | M | M | M | W | W | W | W | W | W |
| L111 | W | M | M | M | W | W | W | W | W | W |
| L198 | W | M | M | M | W | W | W | W | W | W |
| L214 | W | M | M | M | W | W | W | W | W | W |
| L225 | W | M | M | M | W | W | W | W | W | M |
| L231 | W | M | M | M | W | W | W | W | W | W |
