# Supplementary material for: Effect of mass dihydroartemisinin–piperaquine administration in southern Mozambique on the carriage of molecular markers of antimalarial resistance
Source: PLoS One. 2020 Oct 19;15(10):e0240174. doi: 10.1371/journal.pone.0240174 (PMC7571678; doi:10.1371/journal.pone.0240174)
Supplement: S1 Table — (PDF) [file pone.0240174.s002.pdf]

S1 Table. Main characteristics of study participants.

**Variable**		**Pre-MDA (N=99)**	**Post-MDA (N=112)**	**p#**
**Period**		Nov 2015	May 2017	
**Area, n (%)***				
	Magude Sede	65 (68)	59 (53)	0.248
	Motaze	13 (14)	23 (21)	
	Panjane	6 (6)	9 (8)	
	Mahele	3 (3)	7 (6)	
	Mapulanguene	8 (9)	14 (12)	
**Sex, n (%)****				
	Male	47 (48)	53 (48)	1.000
	Female	51 (52)	58 (52)	
**Age (years), mean (SD)*****		14.6 (11.8)	12.8 (17.3)	0.385
**qPCR (parasites/µl), GM (SD)******		90.3 (231.9)	117.4 (371.2)	0.515
**Reported fever in the previous 24 h, n (%)*******				
	Yes	23 (23.2)	39 (34.8)	0.130
	No	70 (70.7)	72 (64.3)	

GM, Geometric mean; SD, Standard deviation

#Student t (continuous) or Fisher exact (categorical) test

* No data for 4 samples (Pre-MDA)

** No data for 2 samples (Pre-MDA=1; Post-MDA=1)

*** No data for 2 samples (Pre-MDA=1; Post-MDA=1)

**** No data for 1 samples (Pre-MDA)

*****No data for 7 samples (Pre-MDA=6; Post-MDA=1)
