# Supplementary material for: Effect of mass dihydroartemisinin–piperaquine administration in southern Mozambique on the carriage of molecular markers of antimalarial resistance
Source: PLoS One. 2020 Oct 19;15(10):e0240174. doi: 10.1371/journal.pone.0240174 (PMC7571678; doi:10.1371/journal.pone.0240174)
Supplement: S2 Table — (PDF) [file pone.0240174.s003.pdf]

S2 Table. Copy number of *pfpm2* and *pfmdr1* in *P. falciparum* isolates from pre- and post-MDA groups.

		**Pre-MDA (n=61)**	**Post-MDA (n=59)**	P value*
***pfpm2* [N(%)]**	≤1.2	46 (75.4)	57 (96.6)	
	1.2-1.5	12 (19.7)	0	1.000
	≥1.5	3 (4.9)	2 (3.4)	
***pfmdr1* [N(%)]**	≤1.2	55 (90.2)	44 (74.6)	
	1.2-1.5	3 (4.9)	12 (20.3)	1.000
	≥1.5	3 (4.9)	3 (5.1)	

**Fisher`s exact test (p value for the comparison of P. falciparum isolates with multiple copies versus the rest)*
